# Supplementary material for: Effect of an E-Prescription Intervention on the Adherence to Surgical Chemoprophylaxis Duration in Cardiac Surgery: A Single Centre Experience
Source: Antibiotics (Basel). 2023 Jul 13;12(7):1182. doi: 10.3390/antibiotics12071182 (PMC10376074; doi:10.3390/antibiotics12071182)
Supplement: Supplementary file 1 [file antibiotics-12-01182-s001.zip › antibiotics-2479146-supplementary.pdf]

SUPPLEMENTARY TABLES

Table S1. Surgical history – pre-operative status (Probably include in Supplementary Data)

	Pre-intervention N (%)	Post -intervention N (%)	p-value
**Patients**	400 (100%)	680 (100%)	
**Procedures**	477	835	
**CABG**	273 (68.3%)	425 (63.5%)	0.056
**AVR**	137 (34.3%)	260 (38.2%)	0.151
**MVR**	42 (10.5%)	86 (10.3%)	0.302
**THOR- AOR**	17 (3.6%)	44 (5.3%)	0.080
**Other combined procedures**	8 (1.7%)	20 (2.4%)	0.392
**Surgical urgency**			0.351
Elective & expedited	266(66.5%)	485(71.3%)	
Urgent	127(31.8%)	186(27.4%)	
Immediate& Salvage	7 (1.8%)	9 (1.3)	
**Duration of surgery, median (interquartile range)**	250 (205 - 295)	230 (185 - 280)	**<0.001***
**Prior CABG/HVR surgery**	67 (16.8%)	118 (17.4%)	0.800
No prior operation	333 (83.3%)	563 (82.8%)	
1 prior operation	58 (14.5%)	89 (13.1%)	
2 prior operations	9 (2.3%)	28 (4.1%)	
**Recent myocardial infraction,** within previous 90 days	231 (57.8%)	333(49.0%)	**0.005**
**Preoperative infection**	15 (3.8%)	27 (4.0%)	0.856
**Preoperative stay ≥ 3days**	196 (49.0%)	268 (39.4%)	0.002
**Preoperative days, median (interquartile range)**	2 (2-4)	2 (2-4)	**<0.001**
**Permanent pacemaker**	11 (2.8%)	24 (3.5%)	0.485
**Endocarditis**	7 (1.8%)	22 (3.2%)	
**Ejection Fraction**			0.364
>50	222 (55.5%)	365 (53.7%)	
31-49	145 (36.3%)	274 (40.3%)	
21-30	30 (7.5%)	37 (5.4%)	
<21	3 (0.8%)	4 (0.6%)	
**Critical preoperative state**	12 (3.0%)	13 (1.9%)	0.250
**NYHA class**			0.010
I	33 (8.3%)	67 (9.9%)	
II	268 (67.2%)	466 (68.5%)	
III	83 (20.8%)	141 (20.7%)	
IV	15 (3.8%)	6 (0.9%)	

CABG: coronary artery bypass graft, **AVR:** Aortic valve replacement, **MVR:** Mitral valve replacement, **THOR- AOR :** Thoracic aorta procedure**, NYHA**: New York heart association
*p value for Mann-Whitney test

Table S2: Risk scores in the pre- and post-intervention cohorts.

Risk score	Pre-intervention N (%)	Post -intervention N (%)	p-value
**ASA score**			<0.001
II	69 (17.3%)	196 (28.9%)	
III	252 (63.0%)	290 (42.7%)	
IV	79 (19.8%)	193 (28.4%)	
**NNIS**			0.113
0	0 (0.0%)	4 (0.6%)	
1	308 (77.0%)	552 (81.3%)	
2	87 (21.8%)	116 (17.1%)	
3	5 (1.3%)	7 (1.0%)	
**EUROSCORE II**			0.778
≤ 3	241 (60.3%)	418(61.5%)	
3 to ≤ 9	114 (28.5%)	196(28.8%)	
10 to ≤ 25	38 (9.5%)	59(8.7%)	
> 25	8 (1.8%)	7(1%)	
**EUROSCORE II, median (interquartile range)**	2.365(1.295-4.6)	2.24(1.275-4.46)	0.579
**CLEVELAND risk score**			0.530
≤ 4	218(54.5%)	377(55.4%)	
4.1 to 10	123(30.8%)	218(32.1%)	
> 10	59(14.8%)	84(12.4%)	
**CLEVELAND median (interquartile range)**	4(2-8)	4(2-8)	0.548

Table S3 Adherence to appropriate PAP duration by surgeon and phase of the study.

Surgeon	PRE N (%)	POST N (%)	p	TOTAL
A	3/57 (5.3%)	38/142 (26.8%)	0.001	41/199 (20.6%)
B	2/14 (14.3%)	1/46 (2.2%)	0.069	3/60 (5.0%)
C	3/53 (5.7%)	18/115 (15.7%)	0.069	21/168 (12.5%)
D	4/185 (2.2%)	40/198 (20.2%)	<0.001	44/383 (11.5%)
E	2/70 (2.9%)	7/177 (4.0%)	0.678	9/247 (3.6%)

Table S4 Univariate analysis of quantitative variables possibly associated with adherence to PAP duration.

Variable	Median, IQR	Median, IQR	P value
Age	67, (59-74)	66, (59-75)	0.73
BMI	26.8, (24.4-29.4)	26.5, (24.5-29.1)	0.67
Preoperative CRP (mg/L)	0.245, (0.1-0.7)	0.26, (0.1-0.72)	0.74
Preoperative WBC (10^9/L)	7.43, (6.17-8.98)	7.81, (6.42-8.89)	0.34
Duration of preoperative hospitalization (days)	2.0, (2.0-4.0)	3.0, (2.0-4.0)	0.11
EUROSCORE II risk score	2.26, (1.28-4.62)	2.43, (1.32-4.04)	0.88
CLEVELAND risk score	4, (2-8)	4, (2-8)	0.99
Duration of operation (min)	240, (195-290)	205, (170-235)	<0.001

IQR: Interquartile range

Table S5 Binary logistic regression for surgical site infection risk factors.

Variable	Odds ratio	Lower 95% C.I.	Upper Lower 95% C.I.	*p*
CLEVELAND category	1.455	0.942	2.248	0.091
Diabetes mellitus	2.748	1.516	4.983	0.001
Neoplasm history	2.710	0.980	7.488	0.055
NYHA	1.637	0.948	2.826	0.077
Preoperative hospitalization ≥ 3 days	1.663	0.925	2.990	0.089
Sex	2.396	1.318	4.355	.004
Phase of the study	2.571	1.262	5.238	.009
